# Plastoglobule-Targeting Competence of a Putative Transit Peptide Sequence from Rice Phytoene Synthase 2 in Plastids

**DOI:** 10.3390/ijms18010018

**Published:** 2016-12-22

**Authors:** Min Kyoung You, Jin Hwa Kim, Yeo Jin Lee, Ye Sol Jeong, Sun-Hwa Ha

**Affiliations:** 1 Department of Genetic Engineering and Graduate School of Biotechnology, College of Life Sciences, Kyung Hee University, Yongin-si 17104, Korea; minkyou@khu.ac.kr (M.K.Y.); yeojin@khu.ac.kr (Y.J.L.); 2 Crop Biotech Institute, Kyung Hee University, Yongin-si 17104, Korea; flowerjin@nate.com (J.H.K.); yesol@khu.ac.kr (Y.S.J.)

**Keywords:** phytoene synthase, plastid, plastoglobule, protoplast, rice, transit peptide

## Abstract

Plastoglobules (PGs) are thylakoid membrane microdomains within plastids that are known as specialized locations of carotenogenesis. Three rice phytoene synthase proteins (OsPSYs) involved in carotenoid biosynthesis have been identified. Here, the N-terminal 80-amino-acid portion of OsPSY2 (PTp) was demonstrated to be a chloroplast-targeting peptide by displaying cytosolic localization of OsPSY2(ΔPTp):mCherry in rice protoplast, in contrast to chloroplast localization of OsPSY2:mCherry in a punctate pattern. The peptide sequence of a PTp was predicted to harbor two transmembrane domains eligible for a putative PG-targeting signal. To assess and enhance the PG-targeting ability of PTp, the original PTp DNA sequence (*PTp*) was modified to a synthetic DNA sequence (*stPTp*), which had 84.4% similarity to the original sequence. The motivation of this modification was to reduce the GC ratio from 75% to 65% and to disentangle the hairpin loop structures of *PTp*. These two DNA sequences were fused to the sequence of the synthetic green fluorescent protein (sGFP) and drove GFP expression with different efficiencies. In particular, the RNA and protein levels of *stPTp-sGFP* were slightly improved to 1.4-fold and 1.3-fold more than those of sGFP, respectively. The green fluorescent signals of their mature proteins were all observed as speckle-like patterns with slightly blurred stromal signals in chloroplasts. These discrete green speckles of PTp*-*sGFP and stPTp*-*sGFP corresponded exactly to the red fluorescent signal displayed by OsPSY2:mCherry in both etiolated and greening protoplasts and it is presumed to correspond to distinct PGs. In conclusion, we identified PTp as a transit peptide sequence facilitating preferential translocation of foreign proteins to PGs, and developed an improved *PTp* sequence, a s*tPTp*, which is expected to be very useful for applications in plant biotechnologies requiring precise micro-compartmental localization in plastids.

## 1. Introduction

Plastoglobules (PGs) are lipid droplets enveloped by a monolayer membrane structure that are attached to chloroplast thylakoid membranes [1]. Several prenylquinone metabolites (tocopherol, phylloquinone, and plastoquinone) have been found in PGs [2], and protein profiling has identified that metabolic enzymes such as ζ-carotene desaturase, lycopene β-cyclase, and β-carotene hydroxylase exist in PG membranes [3,4]. These reports have suggested that PGs play active roles not only in the biosynthesis of tocopherol and other isoprenoid-derived lipid metabolites as versatile lipoprotein particles in plastids, but also in channeling these metabolites in both directions through physical connections to the thylakoid membrane, during plastid development and under various environmental conditions [5,6,7,8]. Studies of the relationship between PGs and carotenoid metabolism in β-carotene–containing sweet orange flesh and rice embryogenic callus have shown that the extent of carotenoid accumulation is strongly associated with the increase in PG number and the abundance of PG-localized proteins [9,10]. PGs have been also proposed as a useful subcellular structure for targeting recombinant proteins within the chloroplast [11]. However, despite the significance of PG localization for metabolic and protein engineering, the transit peptide sequences which act as PG-targeting signals remain largely uncharacterized.

Carotenoids are crucial for photosynthesis, photoprotection, development, and biosynthesis of stress hormones and volatile compounds in plants, and are also prominent metabolites in plant biotechnology [12]. The vital roles of carotenoids as provitamin A components have made them attractive targets of metabolic engineering efforts aimed at improving the nutritional value of various crops [12]. The enzymatic machinery of the carotenoid biosynthetic pathway resides in the plastid envelope, thylakoid membrane, and PGs [13]. Phytoene synthase (PSY), which catalyzes the condensation of two geranylgeranyl pyrophosphate molecules to produce 15-*cis*-phytoene as the first committed enzyme in the carotenoid biosynthesis pathway, has been successfully engineered in several major crop systems to modify carotenoid content as the rate-limiting regulatory enzyme for carotenoid biosynthesis [14,15,16]. One study in maize protoplasts reported that all three rice PSYs were localized to PGs, whereas the three maize PSYs were differentially present in PGs or in the stroma and thylakoid membranes [17].

Based on these results, we reasoned that the three rice PSYs would be promising candidates for identifying PG-targeting transit peptide (Tp) sequences. We predicted the putative Tp sequence and transmembrane (TM) domain of each PSY, and chose an N-terminal 80-amino-acid sequence (PTp) of rice PSY2 (OsPSY2) and its modified sequence (stPTp) for this study. We then evaluated the ability of each sequence to mediate the translocation of a synthetic green fluorescent fusion protein (sGFP) into PGs in a rice protoplast system. We also determined whether DNA modification of PTp to stPTp influenced the expression efficiency of sGFP, both at the RNA and protein levels.

## 2. Results

### 2.1. Bioinformatic Prediction of Putative Plastoglobule-Targeting Sequences from OsPSY1, 2, and 3

Based on previous reports that the three rice PSYs (OsPSY1 (Os06g51290), OsPSY2 (Os12g43130), and OsPSY3 (Os09g38320)) were translocated into PGs in maize protoplasts [5,8,17], we analyzed the amino acid sequences of all three PSYs for putative chloroplast-targeting peptide (Tp) sequences and transmembrane (TM) domains (Table 1) using a bioinformatics approach. The initial prediction of Tp sequences using the ChloroP 1.1 Server (available online: http://www.cbs.dtu.dk/services/ChloroP/) indicated that the N-terminal putative Tp sequences of the three rice PSY proteins had lengths of 21 aa for OsPSY1, 80 aa for OsPSY2, and 54 aa for OsPSY3 (Table 1).

Second, considering that PGs are monolayer membrane structures derived from thylakoid membranes, we predicted putative TM domains using three public databases: TMpred (available online at http://www.ch.embnet.org/software/TMPRED_form.html), TopPred (available online at http://mobyle.pasteur.fr/cgi-bin/portal.py), and HMMTOP (available online at http://www.enzim.hu/hmmtop/index.php). As summarized in Table 1, these databases produced similar results regarding the number and position (i.e., amino acid residue in the full-length protein) of the TM domains. OsPSY1 was predicted to harbor two TM domains in the region encompassing aa 247–267 by all three programs; TMpred and HMMTOP additionally predicted a TM domain at aa 271–293, and TopPred predicted a TM domain at aa 66–86. OsPSY2 was predicted to possess three TM domains at aa 1–22, 46–66, and 233–253 by two programs (TMpred and TopPred); one TM domain at aa 261–278 by HMMTOP; and a single TM domain at 230–252 by all three programs (highlighted in bold). OsPSY3 harbored only one TM domain predicted in the same position, 264–287, by all three software programs.

Interestingly, the first two putative TM domains of OsPSY2 were located at aa 1–22 and 46–66, both of which are within the 80 residues of the putative transit peptide, whereas OsPSY1 and OsPSY3 did not harbor any putative TM domains in their transit peptide regions. Since we assumed that a transit peptide targeting cargo to PGs should harbor both a chloroplast-targeting sequence and TM domains eligible for potentiality of a PG-targeting sequence, OsPSY2 was regarded as the most promising candidate satisfied by the two eligibility criteria for identifying a PG-targeting sequence, in that the OsPSY2 transit peptide (PTp) was predicted to harbor two TM domains in its predicted chloroplast-targeting sequence (80 aa). Thus, we analyzed the potential of the PTp transit peptide as a PG-targeting sequence in this study.

### 2.2. Subcellular Localization of OsPSY2 in Rice Protoplasts

**Figure 1:**
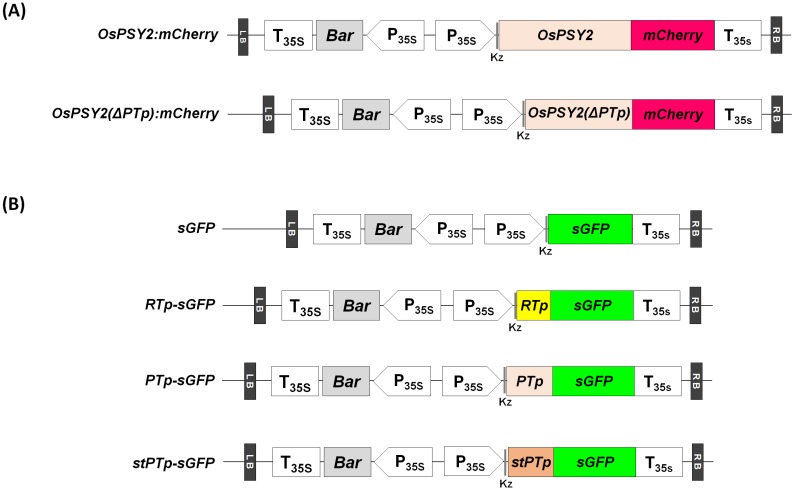
Schematic representation of the sGFP and mCherry fusion constructs transfected into rice protoplasts. (**A**) The red fluorescent mCherry protein was fused to the C-terminus of the full-length OsPSY2 and OsPSY2 (ΔPTp) proteins. OsPSY2, *O. sativa* phytoene synthase 2; PTp, putative transit peptide of OsPSY2; OsPSY2 (ΔPTp), PTp-truncated OsPSY2; (**B**) A synthetic green fluorescent protein (sGFP) was fused to the C-terminus of the RTp-, PTp-, and stPTp-targeting peptides prior to subcellular localization analysis. RTp, transit peptide of the RuBisCO small subunit shown as a yellow color; stPTp, codon-optimized synthetic PTp shown as an orange color. All fusion molecules were expressed under the control of the 35S promoter using the pB2GW7 Gateway plant expression vector. All OsPSY2-originated sequences are shown as a beige color.

**Figure 2:**
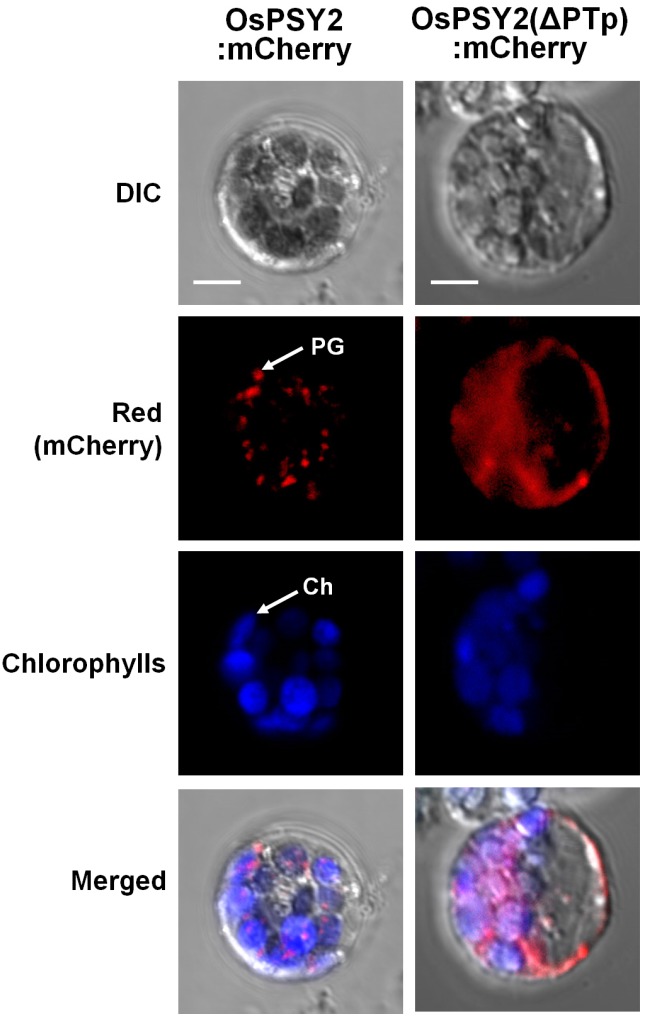
Subcellular localization of OsPSY2:mCherry and OsPSY2(ΔPTp):mCherry. The two mCherry fusion constructs were transfected into rice greening protoplasts of 10-day-old seedlings. Images were acquired at 630× magnification using a confocal microscope. The violet signals resulted from overlaying the red mCherry fluorescence signal with the chlorophyll autofluorescence in the chloroplasts. OsPSY2, full-length OsPSY2 (398 aa); OsPSY2(ΔPTp), truncated form of OsPSY2 lacking the 80 aa transit peptide (318 aa); Ch, chloroplast; PG, plastoglobule. Scale bars = 5 μm.

To ascertain that OsPSY2 localizes to PGs in rice protoplasts and to determine whether PTp functions as a transit peptide for rice phytoene synthase 2 (OsPSY2), the full-length open reading frame (ORF) of OsPSY2 and a truncated form of OsPSY2 lacking the PTp region (ΔPTp) were fused to the N-terminus of a red fluorescent mCherry protein, thereby generating OsPSY2:mCherry and OsPSY2(ΔPTp):mCherry, respectively (Figure 1A). Analysis of the red fluorescent signal of the fusion proteins in rice greening protoplasts revealed that OsPSY2:mCherry exhibited a speckle pattern similar to the localization pattern of OsPSY2 in maize [17], indicating that OsPSY2 localizes to PGs in rice protoplasts as it does in maize (Figure 2). In contrast, OsPSY2(ΔPTp):mCherry showed a pattern typical of cytosol localization, indicating that removal of the N-terminal 80 amino acids from OsPSY2 disrupts the targeting of OsPSY2 to PGs and chloroplasts (Figure 2). This finding suggests that the PTp sequence in OsPSY2 plays an important role in targeting OsPSY2 to chloroplasts (a prerequisite of PG localization) in rice protoplasts, demonstrating its first function as a leader peptide for targeting cargo to PGs. We subsequently endeavored to examine its ability to mediate translocation to PGs specifically.

### 2.3. Modification of the PTp DNA Structure for Improved Applications in Plant Biotechnology

In the process of evaluating the ability of PTp to function as a PG-targeting peptide, we encountered technical challenges in the polymerase chain reaction (PCR) amplification of its coding sequence. We hypothesized that the high GC content (75%) of the PTp DNA sequence (*PTp*) was hindering the amplification reaction. The DNA structure of this sequence was predicted to have five high-scoring secondary structures by a secondary structure prediction program (available online: http://rna.urmc.rochester.edu/RNAstructureWeb/Servers/Predict1/Predict1.html). A high GC ratio and complex secondary structures have been proposed to be the primary factors underlying problematic PCR amplification (Figure S1A) [18]. We thus performed PCR amplification of the PTp DNA sequence in a special GC buffer, and it yielded successful amplification. In addition, we exploited alternate codon usage to modify the *PTp* sequence to a synthetic *PTp* (*stPTp*) sequence, which retained 84.4% DNA similarity and the same amino acid sequence as the original sequence. With these modifications, the high-tension structure of the *PTp* sequence resulting from its five hairpin loops was detangled, and its high GC ratio (75%) was reduced to 65% in the *stPTp* sequence (Figure S1B). As a consequence of these changes, stPTp-DNA fragments were successfully amplified under normal high-fidelity PCR conditions.

**Figure 3:**
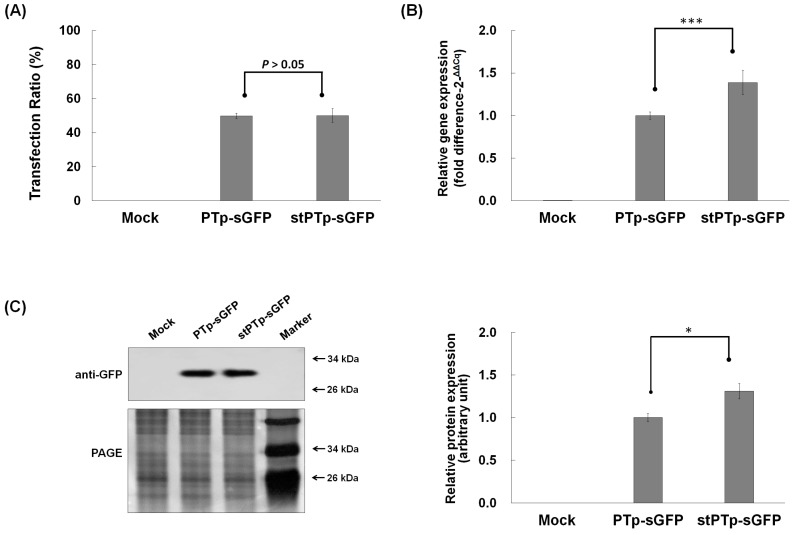
Comparison of PTp-sGFP and stPTp-sGFP expression levels. (**A**) Transfection ratios were determined by measuring the green fluorescent signals of protoplast cells expressing sGFPs. After mounting a 10 μL sample of the transformed protoplasts on a hemocytometer, images were obtained using the DIC and FITC-A channels of a confocal microscope (200× magnification). The error bars show the SEM (standard error of the mean) of data acquired from three independent transfections. Mock samples were prepared by polyethylene glycol (PEG) transfection with no plasmid DNAp; (**B**) Quantitative real-time PCR analysis of sGFP transcript levels in rice protoplasts expressing PTp-sGFP or stPTp-sGFP. The rice ubiquitin gene (AK061988) was used as an internal reference for quantitative normalization; (**C**) Western blot analysis of sGFP protein levels in rice protoplasts expressing PTp-sGFP or stPTp-sGFP. A polyclonal rabbit antibody against GFP was used at a dilution of 1:5000. The silver-stained PAGE gel image shows the relative quantities of the loaded proteins. Luminescent signals were detected and imaged using an LAS4000 system (**left** **panel**), and the relative band intensities were quantified and analyzed using the LAS4000 image analysis program (**right** **panel**). Data are presented as average values of triplicate experiments; error bars denote standard error. * *p* < 0.05, *** *p* < 0.0001.

Additionally, we investigated whether this DNA sequence modification affected gene expression efficiency. After independently transfecting two synthetic green fluorescent protein (sGFP) fusion constructs, *PTp-sGFP* and *stPTp-sGFP*, into rice protoplasts, we measured the transfection ratios and quantified the total RNA and protein expression levels by qRT-PCR and Western blot analysis, respectively (Figure 3). Although the transfection efficiencies of PTp-sGFP (49.8%) and stPTp-sGFP (50.1%) were similar (Figure 3A), the *stPTp* sequence drove higher GFP expression on both the mRNA (1.4-fold) and protein (1.3-fold) levels than the *PTp* sequence (Figure 3B,C). This finding indicates that the modification of the *PTp* sequence to the *stPTp* sequence improves the fusion protein expression on both the transcriptional and translational levels.

### 2.4. Subcellular Localization of sGFP after N-Terminal Fusion of the PTp and stPTp Sequences

**Figure 4:**
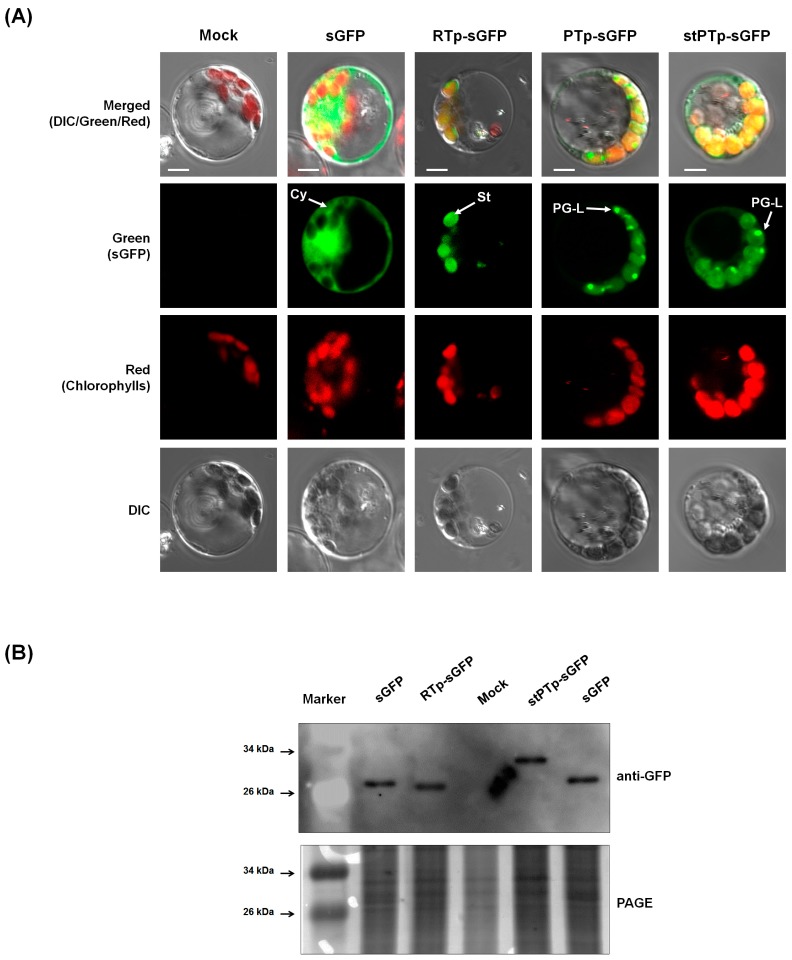
Subcellular localizations of PTp-sGFP and stPTp-sGFP. (**A**) Four sGFP expression constructs (*sGFP*, *RTp-sGFP*, *PTp-sGFP*, and *stPTp-GFP*) were transfected into rice greening protoplasts of 10-day-old seedlings. The images were acquired at 630× magnification using a confocal microscope. The sGFP and RTp-sGFP constructs were used as controls for cytosol and chloroplast stroma localization, respectively. All yellow signals on the merged images were derived from overlap of the green sGFP fluorescence signal and the red chloroplast chlorophyll fluorescence. PEG-transformed protoplasts harboring no plasmids were used as a mock sample. Scale bars = 5 μm; (**B**) Comparison of truncated protein sizes after translocation into chloroplasts. The *sGFP, RTp-sGFP*, and *stPTp-sGFP* constructs were transfected into greening protoplasts of 10-day-old rice seedlings, and 10^6^ cells were used for Western blot analysis. A polyclonal rabbit antibody against GFP was used at a dilution of 1:5000. Luminescent signals were detected and imaged using an LAS4000 system (**upper** **panel**). The silver-stained image of the PAGE gel shows the relative quantities of the loaded proteins. Cy, cytosol; St, stroma; PG-L, PG-like speckles.

To determine the subcellular locations of sGFP fused to the PTp and stPTp peptides, two sGFP fusion molecules, *PTp-sGFP* and *stPTp-sGFP*, were constructed (Figure 1B) and individually transfected into rice greening protoplasts. In a previous study [19], the transit peptide of the ribulose-1,5-bisphosphate carboxylase/oxygenase (RuBisCO) small subunit (RTp) was fused to sGFP, thereby generating RTp-sGFP. We used this construct as a control chloroplast-targeting sequence. The green fluorescent signals from the RTp-sGFP proteins were observed in the chloroplast stroma in a pattern similar to that in the red channel, which is indicative of chlorophyll (Figure 4A). In contrast to their localization patterns, the green fluorescent signals of PTp-sGFP and stPTp-sGFP were observed as strong speckle-like spots with pale signals dispersed over the stroma within chloroplasts. The punctate localization patterns of PTp-sGFP and stPTp-sGFP were identical (Figure 4A) and were quite similar to the PG localization patterns of OsPSY2:mCherry (Figure 2), supposing that PTp-sGFP and stPTp-sGFP might localize to PGs. A previous report showed that the cleaved peptide length of OsPSY2 during chloroplast targeting was shorter than the 80 aa Tp predicted by ChloroP in an in vitro import analysis system using pea chloroplasts [20]. We thus hypothesized that a PG-targeting signal sequence was present in addition to the chloroplast-targeting signal in the PTp and stPTp sequences. To test this hypothesis, we analyzed sGFP, RTp-sGFP, and stPTp-sGFP by Western blotting. As shown in Figure 4B, the RTp of RTp-sGFP was completely cleaved to 27.1 kDa (the original sGFP size) after translocation into chloroplasts, whereas the synthetic PTp (stPTp, 80 aa) of stPTp-sGFP was only partially cleaved, thereby generating a protein larger (~30 kDa) than the original sGFP. This finding suggests that only part (~50 aa) of the 80-amino-acid sequence predicted by ChloroP is cleaved during chloroplast translocation, whereas the remainder (~30 aa) harbors the PG-targeting signal within chloroplasts.

**Figure 5:**
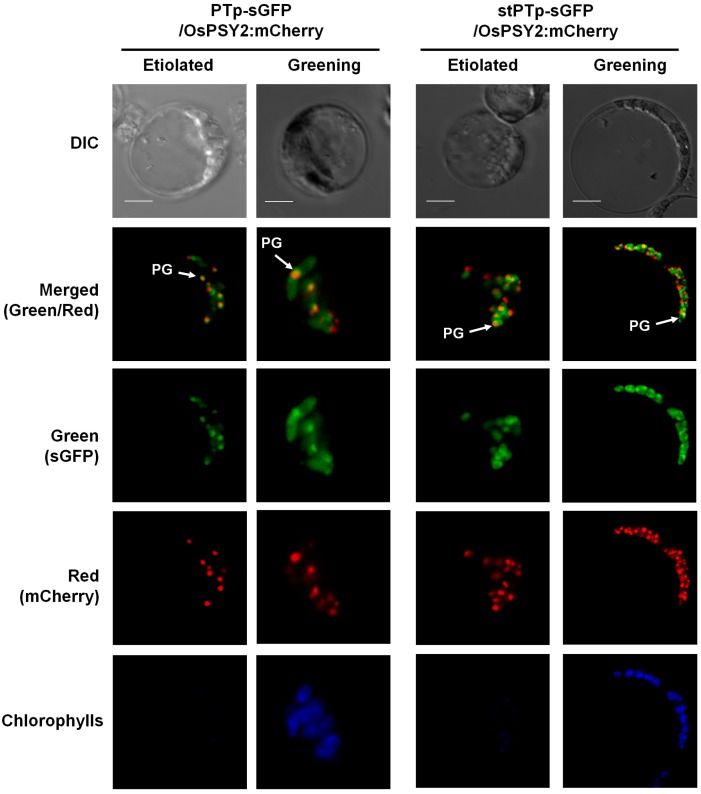
Subcellular localizations of OsPSY2:mCherry, PTp-sGFP, and stPTp-sGFP. The OsPSY2:mCherry construct was co-transfected with PTp-sGFP or stPTp-sGFP into rice etiolated or greening protoplasts isolated from 10-day-old seedlings. Images were acquired at 630× magnification using a confocal microscope. The yellow signals correspond to merged images of the green fluorescent signals from sGFP and the red fluorescent signals from mCherry. Blue fluorescence indicates chlorophyll in the chloroplast. Scale bars = 5 μm. PG, plastoglobule.

To determine whether the speckle-like green signals of PTp-sGFP and stPTp-sGFP within chloroplasts corresponded to PGs, OsPSY2:mCherry was co-transfected with either PTp-sGFP or stPTp-sGFP into rice etiolated and greening protoplasts containing two different kinds of plastids, etioplasts or chloroplasts, respectively. The rationale behind this approach was that PGs are considered to be closely associated with plastid differentiation [8]. We found that the discrete green speckles of PTp*-*sGFP and stPTp*-*sGFP matched exactly with the red fluorescent signal of OsPSY2:mCherry, presumably reflecting distinct PG particles. Specifically, the overlap was visualized as yellow speckles after merging the green and red channels in chlorophyll areas (Figure 5). However, the green fluorescence signals of PTp-sGFP and stPTp-sGFP were also observed in the stromal area, in addition to the speckle-like patterns presumed to be PGs, in contrast to the red fluorescence signals of OsPSY2:mCherry clearly observed on PGs. These findings indicated that most PTp/stPTp-sGFP were successfully translocated to PGs within chloroplasts, whereas a minor proportion of them failed to be localized to PGs.

## 3. Discussion

Since the significance of PGs as a biosynthetic venue of isoprenoid-derived lipid metabolites (including carotenoids) and as a reservoir of these metabolites was established, PGs have been considered to be useful subcellular structures within plastids for metabolic and protein engineering [11]. Considering the limited knowledge of the Tp sequences used for PG targeting, carotenogenic enzymes, which are PG-localized proteins, are a potentially useful source of Tp sequences for facilitating more sophisticated plant biotechnology.

In this study, a putative PTp sequence (80 aa) derived from OsPSY2 was identified as a potential PG-targeting peptide by bioinformatics analysis predicting two putative TM domains within the transit peptide regions; sequences meeting the same characteristics were not found in OsPSY1 and OsPSY3 (Table 1). The PG-targeting abilities of the PTp and stPTp sequences were examined using sGFP in greening rice protoplasts (Figure 4), and the green fluorescent signals were similar to speckle-like spots with pale signals dispersed over the stroma (Figure 2). To determine whether these punctate signals corresponded to PGs, cotransfection experiments were performed with OsPSY2:mCherry. The green speckles observed with PTp-sGFP or stPTp-sGFP corresponded exactly to the red speckles observed with OsPSY2:mCherry, as demonstrated by the yellow spots in Figure 5. As shown in Figure 4B, the full size of PTp-sGFP/stPTp-sGFP is 35.6 kDa, and the truncated stPTp-sGFP is larger (~30 kDa) than sGFP (27.1 kDa). This finding suggests that the 80 aa Tp sequence of OsPSY2 (PTp/stPTp), which was predicted to be a plastid-targeting peptide via ChloroP, consists of two parts. The first part (~50 aa) is required for targeting to chloroplasts, and the other part (~30 aa) remains at the N-terminus of sGFP in chloroplasts even after translocation. These two properties of PTp/stPTp are presumably responsible for the different fluorescence signals obtained with the stroma-targeting RTp-sGFP proteins (Figure 4A) and the similar fluorescence signals obtained with the OsPSY2:mCherry PG-targeting proteins (Figure 5). Hence, we suggest that the 80 aa PTp sequence originating from OsPSY2 has two important functions for targeting to chloroplasts as well as preferentially to PGs within chloroplasts. Considering the dispersed stromal signals of PTp-sGFP and stPTp-sGFP (Figure 2 and Figure 5), we hypothesize that additional signals might be required for PG-targeting. Such signals might be related to the positions of the TM domains in Table 1; specifically, PG-targeting signals might be closely related to the second and third TM domains of OsPSY2. These hypotheses should be examined in future studies to further elucidate the PG-targeting mechanism of OsPSY2.

In addition to evaluating the potential of PTp/stPTp as PG-targeting transit peptides, the PG localization of OsPSY2 previously reported in maize [17] was also verified in rice (Figure 2). Also, to overcome the structural weaknesses of the *PTp* sequence in the context of plant biotechnology applications, the sequence was modified within the allowable range of rice codon usages, thereby generating a *stPTp* sequence (Figure S1). The resulting decreased GC content and the fewer hairpin loops of the *stPTp* sequence contributed to the enhanced expression of GFP on both the RNA and protein levels (Figure 3). Furthermore, the PCR amplification efficiency improved.

In summary, we reported a PG-preferential Tp sequence originating from OsPSY2, a key carotenoid enzyme. Considering the importance of PGs in the biosynthesis and accumulation of carotenoids, tocopherols, and lipids, and the value of PGs as a new location for foreign recombinant proteins requiring elaborate sequestration into PGs [11], the PTp/stPTp sequence is anticipated to have useful applications in protein functional analysis, plant molecular farming, and plant metabolic engineering of processes occurring in PGs.

## 4. Materials and Methods

### 4.1. Vector Construction

The *mCherry* gene was amplified from pmCherry-C1 (Clontech, Tokyo, Japan) using *Not*I-*mCherry*-fwd/*mCherry*-rev primers and then subcloned as *pDONR221-NotI-mCherry* using Gateway^®^ BP Clonase^®^ II Enzyme Mix (Invitrogen, Waltham, MA, USA). The full-length open-reading frame of *OsPSY2* was amplified from rice leaf cDNA pools using *Not*I-*OsPSY2*-fwd/*Not*I-*OsPSY2*-rev primers, digested with *Not*I, and ligated with the digested *pDONR221*-*Not*I-*mCherry* vector, thereby generating *pDONR221-OsPSY2:mCherry*. The PCR amplicon *attB1-OsPSY2(ΔPTp):mCherry-attB2* was introduced into *pDONR221*, thereby producing *pDONR221-OsPSY2(ΔPTp):mCherry*, using Gateway^®^ BP Clonase^®^ II Enzyme Mix (Invitrogen).

The *sGFP* gene was amplified from pSB-RTG [21] using two primer sets, introducing a nonhomologous overhang of a plant Kozak sequence (Kz, AACAATGGC) [22], an *Nde*I restriction enzyme site, and recombination sites (attB1 and attB2) for the Gateway cloning system [23]. The resultant amplicon was cloned into pDONR221 using Gateway^®^ BP Clonase^®^ II Enzyme Mix (Invitrogen), thereby constructing two in-house entry vector backbones of *pDONR221-Kz-Nde*I*-sGFP*. The *PTp* DNA fragments were amplified from cDNA pools prepared from the leaves of two-week-old seedlings using attB1-PTp-fwd/attB2-PTp-rev primers and an mRNA Selective PCR Kit Ver 1.1 (Takara Bio, Shiga, Japan). The modified DNA sequence, *stPTp*, was amplified with *Nde*I-*stPTp*-fwd/*Nde*I-*stPTp*-rev primers using a DNA template synthesized by a custom gene synthesis service (Bioneer, Daejeon, Korea). Chimeric fragments of *PTp-sGFP* with attB1-B2 sites were prepared by overlap extension-PCR [24]. The chimeric gene fragment of *PTp-sGFP* was cloned into pDONR221 using Gateway^®^ BP Clonase^®^ II Enzyme Mix according to the manufacturer’s instructions (Invitrogen). The amplified *Nde*I-*stPTp*-*Nde*I fragment was digested with *Nde*I and ligated into *pDONR221-Kz*-*Nde*I-*sGFP*, thereby generating *pDONR221*-*stPTp-sGFP*.

The resulting four gene fragments, *OsPSY2:mCherry*, *OsPSY2(ΔPTp):mCherry*, *PTp-sGFP*, and *stPTp-sGFP*, were introduced into *pB2GW7* (containing the 35S promoter for constitutive expression) through Gateway cloning [25] with Gateway^®^ LR Clonase^®^ II Enzyme Mix (Invitrogen), as shown in Figure 1. Construction of *sGFP* and *RTp*-*sGFP* was described in a previous study [19]. All PCR primers used in this study are listed in Table S1, and all PCR reactions except those whose templates contained *PTp* sequences were performed using Phusion^®^ High-Fidelity DNA Polymerase (New England Biolabs, Ipswich, MA, USA). All DNA templates containing a *PTp* sequence were amplified under the same PCR conditions using Ex Taq^TM^ or LA Taq^®^ with GC buffer I (Takara Bio).

### 4.2. Protoplast Preparation

Rice (*Oryza sativa* L. *Japonica* cv. “Ilmi”) seeds were sterilized and germinated as described [19], after which the seedlings were grown at 28 °C for 10 days in the dark to produce etiolated protoplasts or under 16 h/8 h (light/dark) conditions to produce greening protoplasts. Rice protoplasts were isolated as previously described [19,26] with minor modifications. Briefly, rice seedling leaf sheaths were cut into 0.5-mm segments with new razor blades and then immediately immersed in 15 mL of Cellulase R-10 and Macerozyme R-10 enzyme solution (Yakult Honsha, Japan) for cell wall digestion. After vacuum infiltration for 10 min to increase digestion efficiency and a further incubation of 4 h with gentle shaking (50 rpm) at RT in the dark, the enzyme reaction was stopped by the addition of 30 mL W5 solution. Cell debris was removed by filtering twice through 70 and 40 μm Falcon™ cell strainers (BD, Franklin Lakes, NJ, USA), and the protoplast pellets were suspended in Mmg buffer solution at 10^7^ protoplasts/mL for PEG transfection [26].

### 4.3. Vector DNA Transfection and Microscopy Analysis

For polyethylene glycol (PEG) transfection, 10^6^ protoplast cells as counted by a Marienfeld hemocytometer counting system (Marienfeld-Superior, Berlin, Germany) were transfected with 5 μg of each plasmid with an equal volume of 40% PEG-3350 solution (Sigma, St. Louis, MO, USA) containing 0.5 M mannitol and 100 mM CaCl_2_. After incubation for 15 min, the protoplasts were washed twice with each of two volumes of W5 and 1 mL of incubation solution, resuspended in 1 mL of incubation solution, and incubated at 28 °C in the dark overnight. All plasticware used for protoplasts was precoated with 5% calf serum by swirling for 10 s, and all buffers were filtered using 0.45 µm syringe filters (Sartorius, Gottingen, Germany).

Fluorescence signals from the transfected protoplasts were observed using a Carl Zeiss LSM700 inverted confocal microscope. Images were acquired using ZEN 2009 Light Edition software (Carl Zeiss, Oberkochen, Germany). sGFP fluorescence was detected with excitation and emission wavelengths of 488 nm and 505–530 nm, respectively; chlorophyll fluorescence was analyzed with excitation and emission wavelengths of 555 and >650 nm, respectively; and mCherry fluorescence was detected with excitation and emission wavelengths of 543 and 610–700 nm, respectively.

### 4.4. Molecular Analysis of sGFP Expression

Transfected protoplasts (10^6^ cells) were pelleted, immediately frozen in liquid N_2_, and stored at −80 °C until RNA and protein extraction. Total RNA was extracted using the RNeasy^®^ Plant Mini kit (Qiagen, Hilden, Germany), and first-strand cDNA was synthesized using an mRNA selective PCR kit (AMV) (ver. 1.1, Takara Bio, Shiga, Japan). Quantitative real-time PCR (qRT-PCR) was performed using Thunderbird™ SYBR^®^ qPCR mix (Toyobo, Osaka, Japan); SYBR-fluorescence signals were detected and quantified using a CFX96™ Real-Time PCR system (Bio-Rad, Foster City, CA, USA). The SYBR fluorescence of the *sGFP* transcript was quantified using CFX Manager™ ver. 2.1 (Bio-Rad). A rice ubiquitin gene (*AK061988*) was used as a reference to normalize the amount of protoplast RNA used [27].

Total protein extracts were also prepared from the same frozen protoplast pellets through suspension in 160 μL of phosphate-buffered saline (PBS; Caisson Laboratories, North Logan, UT, USA) and 40 μL of 5× SDS-PAGE loading buffer (Biosesang, Seongnam, Korea). After boiling for 10 min, the protoplasts were centrifuged at 4 °C, and 5 μL of the supernatant was loaded onto 12% acrylamide gels. Proteins were separated by SDS-PAGE and transferred to PVDF membranes (Whatman, Kent, UK) using a Trans-Blot^®^ SD Semi-Dry Electrophoretic Transfer Cell (Bio-Rad). In parallel, a duplicate gel was stained with the EZ-Silver Staining Kit for Protein (Biosesang) to quantify the amount loaded and assess the quality of the extracts. 

Western blotting was carried out with an anti-rabbit polyclonal antibody against GFP (Abcam, Cambridge, UK) and a secondary anti-rabbit alkaline phosphatase conjugate (Promega, Madison, WI, USA). Both antibodies were used at a dilution of 1:5000. After incubation with Novex^®^ AP Chemiluminescent Substrate CDP-Star^®^ (Invitrogen), immunoreactive bands were imaged and quantified using a LAS-4000 luminescence detector and LAS 4000 software (GE Healthcare Life Sciences, Little Chalfont, UK).

### 4.5. Statistical Analysis

All expression analyses were performed in triplicate. Differences between groups were analyzed using Student’s *t*-test, and *p* values < 0.05 were considered to be significant.

## Figures and Tables

**Table 1:** Transit peptide and transmembrane prediction of rice PSYs. The number of amino acids predicted as putative transit peptide and common regions by all three programs among predicted transmembrane regions are highlighted in bold.

Protein (Size, aa)	ChloroP ^a^	TMpred ^b^				TopPred ^c^				HMMTOP ^d^		
	Tp *	TM ^†^ No.	TM 1	TM 2	TM 3	TM No.	TM 1	TM 2	TM 3	TM No.	TM 1	TM 2
**OsPSY1 (420)**	**21**	2	**247–265**	271–293		2	66–86	**247–267**		2	**249–266**	275–292
**OsPSY2 (398)**	**80**	3	1–22	48–66	**233–252**	3	1–21	46–66	**233–253**	2	**230–252**	261–278
**OsPSY3 (444)**	**54**	1		**264–285**		1	**267–287**			1	**264–285**	

Tp *, transit peptide; TM ^†^, transmembrane; Bold fonts, a common region for all three programs; ChloroP ^a^, available online: http://www.cbs.dtu.dk/services/ChloroP/; TMpred ^b^, available online; http://www.ch.embnet.org/software/TMPRED_form.html; TopPred ^c^, available online: http://mobyle.pasteur.fr/cgi-bin/portal.py; HMMTOP ^d^, available online: http://www.enzim.hu/hmmtop/index.php.

## Supplementary Materials

Supplementary materials can be found at www.mdpi.com/1422-0067/18/1/18/s1.

## References

[1] Greenwood A.D., Leech R.M., Williams J.P. (1963). The osmiophilic globules of chloroplasts: I. Osmiophilic globules as a normal component of chloroplasts and their isolation and composition in *Vicia faba* L. Biochim. Biophys. Acta.

[2] Lichtenthaler H.K. (1969). Die Plastoglobuli. Protoplasma.

[3] Vidi P.A., Kanwischer M., Baginsky S., Austin J.R., Csucs G., Dormann P., Kessler F., Brehelin C. (2006). Tocopherol cyclase (VTE1) localization and vitamin E accumulation in chloroplast plastoglobule lipoprotein particles. J. Biol. Chem..

[4] Ytterberg A.J., Peltier J.B., van Wijk K.J. (2006). Protein profiling of plastoglobules in chloroplasts and chromoplasts. A surprising site for differential accumulation of metabolic enzymes. Plant Physiol..

[5] Brehelin C., Kessler F., van Wijk K.J. (2007). Plastoglobules: Versatile lipoprotein particles in plastids. Trends Plant Sci..

[6] Schweiggert R.M., Steingass C.B., Heller A., Esquivel P., Carle R. (2011). Characterization of chromoplasts and carotenoids of red- and yellow-fleshed papaya (*Carica papaya* L.). Planta.

[7] Davidi L., Levin Y., Ben-Dor S., Pick U. (2015). Proteome analysis of cytoplasmatic and plastidic β-carotene lipid droplets in *Dunaliella bardawil*. Plant Physiol..

[8] Rottet S., Besagni C., Kessler F. (2015). The role of plastoglobules in thylakoid lipid remodeling during plant development. Biochim. Biophys. Acta.

[9] Bai C., Rivera S.M., Medina V., Alves R., Vilaprinyo E., Sorribas A., Canela R., Capell T., Sandmann G., Christou P. (2014). An in vitro system for the rapid functional characterization of genes involved in carotenoid biosynthesis and accumulation. Plant J..

[10] Zeng Y., Du J., Wang L., Pan Z., Xu Q., Xiao S., Deng X. (2015). A comprehensive analysis of chromoplast differentiation reveals complex protein changes associated with plastoglobule biogenesis and remodeling of protein systems in sweet orange flesh. Plant Physiol..

[11] Vidi P.A., Kessler F., Brehelin C. (2007). Plastoglobules: A new address for targeting recombinant proteins in the chloroplast. BMC Biotechnol..

[12] Giuliano G. (2014). Plant carotenoids: Genomics meets multi-gene engineering. Curr. Opin. Plant Biol..

[13] Shumskaya M., Wurtzel E.T. (2013). The carotenoid biosynthetic pathway: Thinking in all dimensions. Plant Sci..

[14] Ye X., Al-Babili S., Kloti A., Zhang J., Lucca P., Beyer P., Potrykus I. (2000). Engineering the provitamin A (β-carotene) biosynthetic pathway into (carotenoid-free) rice endosperm. Science.

[15] Ha S.H., Liang Y.S., Jung H., Ahn M.J., Suh S.C., Kweon S.J., Kim D.H., Kim Y.M., Kim J.K. (2010). Application of two bicistronic systems involving 2A and IRES sequences to the biosynthesis of carotenoids in rice endosperm. Plant Biotechnol. J..

[16] Kim M.J., Kim J.K., Kim H.J., Pak J.H., Lee J.H., Kim D.H., Choi H.K., Jung H.W., Lee J.D., Chung Y.S. (2012). Genetic modification of the soybean to enhance the β-carotene content through seed-specific expression. PLoS ONE.

[17] Shumskaya M., Bradbury L.M., Monaco R.R., Wurtzel E.T. (2012). Plastid localization of the key carotenoid enzyme phytoene synthase is altered by isozyme, allelic variation, and activity. Plant Cell.

[18] Singh V.K., Govindarajan R., Naik S., Kumar A. (2000). The Effect of hairpin structure on PCR amplification efficiency. Mol. Biol. Today.

[19] You M.K., Lim S.H., Kim M.J., Jeong Y.S., Lee M.G., Ha S.H. (2015). Improvement of the fluorescence intensity during a flow cytometric analysis for rice protoplasts by localization of a green fluorescent protein into chloroplasts. Int. J. Mol. Sci..

[20] Welsch R., Wust F., Bar C., Al-Babili S., Beyer P. (2008). A third phytoene synthase is devoted to abiotic stress-induced abscisic acid formation in rice and defines functional diversification of phytoene synthase genes. Plant Physiol..

[21] Jang I.-C., Nahm B.H., Kim J.-K. (1999). Subcellular targeting of green fluorescent protein to protein to plastids in transgenic rice plants provides a high-level expression system. Mol. Breed..

[22] Lutcke H.A., Chow K.C., Mickel F.S., Moss K.A., Kern H.F., Scheele G.A. (1987). Selection of AUG initiation codons differs in plants and animals. EMBO J..

[23] Hartley J.L., Temple G.F., Brasch M.A. (2000). DNA cloning using in vitro site-specific recombination. Genome Res..

[24] Higuchi R., Krummel B., Saiki R.K. (1988). A general method of in vitro preparation and specific mutagenesis of DNA fragments: Study of protein and DNA interactions. Nucleic Acids Res..

[25] Dubin M.J., Bowler C., Benvenuto G. (2008). A modified Gateway cloning strategy for overexpressing tagged proteins in plants. Plant Methods.

[26] Bart R., Chern M., Park C.J., Bartley L., Ronald P.C. (2006). A novel system for gene silencing using siRNAs in rice leaf and stem-derived protoplasts. Plant Methods.

[27] Song M.H., Lim S.H., Kim J.K., Jung E.S., Maria John K.M., You M.K., Ahn S.N., Lee C.H., Ha S.H. (2016). In planta cleavage of carotenoids by Arabidopsis carotenoid cleavage dioxygenase 4 in transgenic rice plants. Plant Biotechnol. Rep..

