# Supplementary material for: Plastoglobule-Targeting Competence of a Putative Transit Peptide Sequence from Rice Phytoene Synthase 2 in Plastids
Source: Int J Mol Sci. 2016 Dec 22;18(1):18. doi: 10.3390/ijms18010018 (PMC5297653; doi:10.3390/ijms18010018)
Supplement: Supplementary file 1 [file ijms-18-00018-s001.pdf]

# Supplementary Materials: Plastoglobule-Targeting Competence of a Putative Transit Peptide Sequence from Rice Phytoene Synthase 2 in Plastids

**Min Kyoung You, Jin Hwa Kim, Yeo Jin Lee, Ye Sol Jeong and Sun-Hwa Ha**

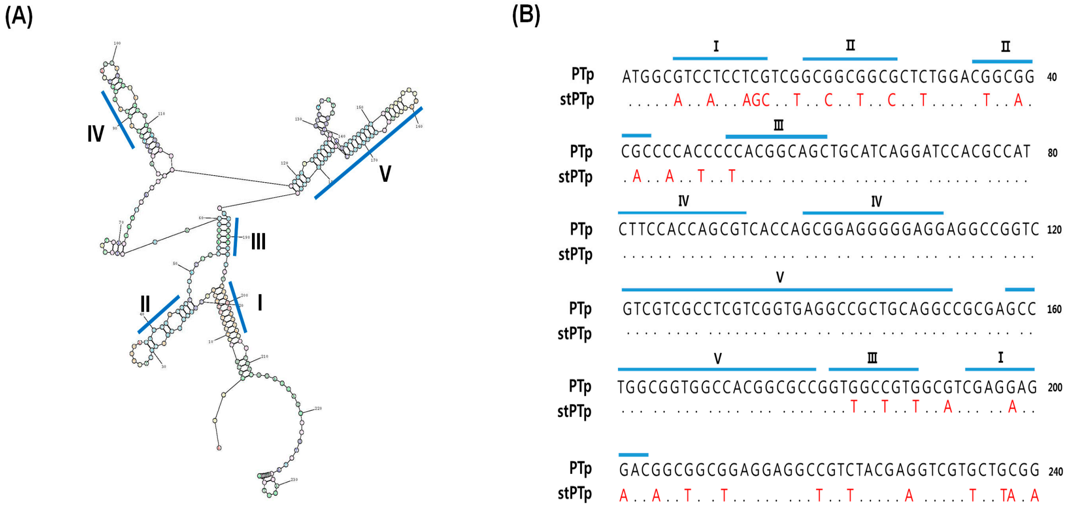


**Figure S1.** Predicted secondary DNA structures of the *PTp* and *stPTp* sequences. (**A**) Possibility of DNA secondary structures in the *PTp* sequence as predicted by a secondary structure prediction server (available online: http://rna.urmc.rochester.edu/RNAstructureWeb/Servers/Predict1/Predict1.html). Parameters included DNA as the nucleic acid type and selection of the MaxExpect method, which is thought to have higher fidelity in structure prediction. (**B**) Alignment of the *PTp* and *stPTp* sequences. The DNA sequences modified in *stPTp* are shown in red. Regions indicated by the five blue lines possess a high probability (>60) of secondary structure in both (**A**) and (**B**).

**Table S1.** List of primers used in this study.

| Primers for Cloning | Sequence (5′-3′) |
|---|---|
| attB1-*Not*I-mCherry-fwd | AAAAAGCAGGCTGCGGCCGCATGGTGAGCAAGGGCGAGGAGGA |
| attB2-mCherry-rev | AGAAAGCTGGGTCTTGTACAGCTCGTCCATGCCGCCGGTG |
| *Not*I-OsPSY2-fwd | AAGCAGGCTGCGGCCGCATGGCGTCCTCCTCGTCGG |
| *Not*I-OsPSY2-rev | ACCATGCGGCCGCCTGATGCAACTGCCGCTCTTGCATAAG |
| attB1-OsPSY2(ΔPTp)-mCherry-fwd | GGGGACAAGTTTGTACAAAAAAGCAGGCTAACAATGGCCCAGGCGGCGCTGGTGGAGGAG |
| attB2-OsPSY2(ΔPTp)-mCherry-rev | GGGGACCACTTTGTACAAGAAAGCTGGGTTTACTTGTACAGCTCGTCCATGC |
| attB1-*Nde*I-sGFP-fwd | AGGCTTCAACAATGGCCCATATGGTGAGCAAGGGCGAGGAGCTGTTCACCGGGGTGGTGCCCATC |
| attB2-sGFP-rev | GGGGACCACTTTGTACAAGAAAGCTGGGTCCTATTACTTGTACAGCTCGTCCATGCCGTG |
| attB1-PTp-fwd | GGGGACAAGTTTGTACAAAAAAGCAGGCTTCAACAATGGCCGCGTCCTCCTCGTCGGCG |
| PTp-sGFP_overlaid-rev | GGTGAACAGCTCCTCGCCCTTGCTCACCATCCGCAGCACGACCTCGTAGACGGCCTCCT |
| PTp-sGFP_overlaid-fwd | GAGGCCGTCTACGAGGTCGTGCTGCGGATGGTGAGCAAGGGCGAGGAGCTGTTCACCGGG |
| attB2-sGFP-rev | GGGGACCACTTTGTACAAGAAAGCTGGGTCCTATTACTTGTACAGCTCGTCCATGCCGTG |
| *Nde*I-stPTp-fwd | GAATTCGATCATATGGCATCATCCAGCTCTGCCG |
| *Nde*I-stPTp-rev | CCGCGAATTCACTAGTGATCATATGGGTACCTC |
| **Primers for qRT-PCR** | **Sequence (5′-3′)** |
| **sGFP chimera transcript** | |
| sGFP-fwd | CTGCCCGACAACCACTACCTGAG |
| sGFP-rev | GGCGGCGGTCACGAACT |
| **Internal reference transcript** | |
| OsUbi (AK061988)-fwd | GAAGTAAGGAAGGAGGAGGA |
| OsUbi (AK061988)-rev | AAGGTGTTCAGTTCCAAGG |
